# Alterations in UPR Signaling by Methylmercury Trigger Neuronal Cell Death in the Mouse Brain

**DOI:** 10.3390/ijms232315412

**Published:** 2022-12-06

**Authors:** Ryosuke Nomura, Nobumasa Takasugi, Hideki Hiraoka, Yuta Iijima, Takao Iwawaki, Yoshito Kumagai, Masatake Fujimura, Takashi Uehara

**Affiliations:** 1 Department of Medicinal Pharmacology, Graduate School of Medicine, Dentistry and Pharmaceutical Sciences, Okayama University, Okayama 700-8530, Japan; 2 Division of Cell Medicine, Department of Life Science, Medical Research Institute, Kanazawa Medical University, Kahoku 920-0293, Japan; 3 Environmental Biology Laboratory, Faculty of Medicine, University of Tsukuba, Tsukuba 305-8575, Japan; 4 Department of Basic Medical Science, National Institute for Minamata Disease, Kumamoto 867-0008, Japan

**Keywords:** methylmercury, neuronal cell death, endoplasmic reticulum stress, unfolded protein response, ERAI system

## Abstract

Methylmercury (MeHg), an environmental toxicant, induces neuronal cell death and injures specific areas of the brain. MeHg is known to induce oxidative and endoplasmic reticulum (ER) stress. The unfolded protein response (UPR) pathway has a dual nature in that it regulates and protects cells from an overload of improperly folded proteins in the ER, whereas excessively stressed cells are eliminated by apoptosis. Oxidative stress/ER stress induced by methylmercury exposure may tilt the UPR toward apoptosis, but there is little in vivo evidence of a direct link to actual neuronal cell death. Here, by using the ER stress-activated indicator (ERAI) system, we investigated the time course signaling alterations of UPR in vivo in the most affected areas, the somatosensory cortex and striatum. In the ERAI-Venus transgenic mice exposed to MeHg (30 or 50 ppm in drinking water), the ERAI signal, which indicates the activation of the cytoprotective pathway of the UPR, was only transiently enhanced, whereas the apoptotic pathway of the UPR was persistently enhanced. Furthermore, detailed analysis following the time course showed that MeHg-induced apoptosis is strongly associated with alterations in UPR signaling. Our results suggest that UPR modulation could be a therapeutic target for treating neuropathy.

## 1. Introduction

Methylmercury (MeHg), a causative factor of Minamata disease, is an environmental material with potent neurotoxicity. MeHg is derived from inorganic mercury released into the atmosphere and deposited in the ocean due to various sources such as volcanic activity and gold mining. Inorganic mercury is transformed into MeHg by microorganisms and accumulates in edible fish such as tuna through bioconcentration. Therefore, the main source of MeHg exposure in humans is the consumption of these fish [1]. It has been reported that MeHg easily crosses the blood–brain barrier and shows a specific loss of neurons in the brain [2,3]. In patients with Minamata disease, MeHg mainly injures the cerebellum and cerebral cortex, including the postcentral gyrus, Heschl’s gyrus, and calcarine sulcus [4]. Intriguingly, the involvement of MeHg in the pathogenesis of neurodegenerative diseases such as Alzheimer’s disease (AD) and Parkinson’s disease (PD) has been reported [4,5]. However, the detailed mechanisms of site- and cell-specific neurotoxicity caused by MeHg remain to be fully elucidated.

Several reports suggest that endoplasmic reticulum (ER) stress is induced by MeHg exposure [6,7,8,9]. Endoplasmic reticulum stress is caused by various abnormal conditions such as the accumulation of misfolded proteins, depletion of Ca^2+^ stores in the endoplasmic reticulum, and oxidative stress [10]. MeHg has a high affinity for nucleophiles and causes protein dysfunction through covalent binding to sulfhydryl groups in the cysteine residues of proteins (S-mercuration) [11]. Some of the regulators of ER stress have been shown to be targets of MeHg. In previous reports, manganese superoxide dismutase (MnSOD) and glutathione (GSH) were thought to contribute to MeHg toxicity by causing oxidative stress due to the suppression of their functions by S-mercuration [12,13]. It has also been shown that ER stress occurs after oxidative stress induction by MeHg, causing cytotoxicity [6,8], and that misfolded proteins accumulate in the ER due to the reduction in protein disulfide isomerase (PDI) chaperone activity caused by S-mercuration [14].

Cells sensing ER stress maintain intracellular protein homeostasis by the unfolded protein response (UPR) [15]. The UPR consists of three representative pathways mediated by ER membrane proteins: inositol-requiring enzyme 1α (IRE1α), protein kinase RNA-like ER kinase (PERK), and activating transcription factor 6 (ATF6). Under mild ER stress, the cytoprotective UPR signals work, such as the suppression of protein translation, promoting the induction of molecular chaperones and components of ER-associated degradation (ERAD). In contrast, under excessive ER stress, cytotoxic UPR signals work to eliminate damaged cells, the UPR induces the expression of C/EBP homologous protein (CHOP), a major factor in ER stress-mediated apoptosis, and leads to apoptosis. Thus, the UPR functions in both cell survival and cell death, but the mechanism by which it switches from the protective pathway to the apoptotic pathway is unknown.

When cells sense ER stress, IRE1α self-activates by autophosphorylation and splices X-box binding protein 1 (XBP1) mRNA independent of the spliceosome. We previously showed that electrophiles such as MeHg and nitric oxide decrease XBP1 mRNA splicing and induce apoptosis in vitro [16,17]. Additionally, UPR dysfunction has been suggested to contribute to the onset of neurodegenerative diseases [18]. Therefore, we predicted that induction of ER stress and alteration of UPR function by MeHg exposure may contribute to the neurodegeneration in such as Minamata disease. However, the relationship between MeHg exposure and functional alterations of the UPR has not been investigated in vivo.

In this study, we used ER stress-activated indicator (ERAI) transgenic mice (ERAI-Venus mice) [19]. Under ER stress conditions, the XBP1 region of the ERAI gene is spliced by IRE1α, which causes a frameshift resulting in the expression of XBP1 fused to Venus. Using this mouse model, we can monitor the site-specific occurrence of ER stress in vivo by immunohistochemistry. We analyzed whether MeHg-induced ER stress and functional alterations in the UPR pathways contribute to neuronal cell death.

## 2. Results

### 2.1. ERAI-Venus Mice Exhibit Neurological Symptoms Similar to Wild-Type Mice upon MeHg Exposure

**Figure 1:**
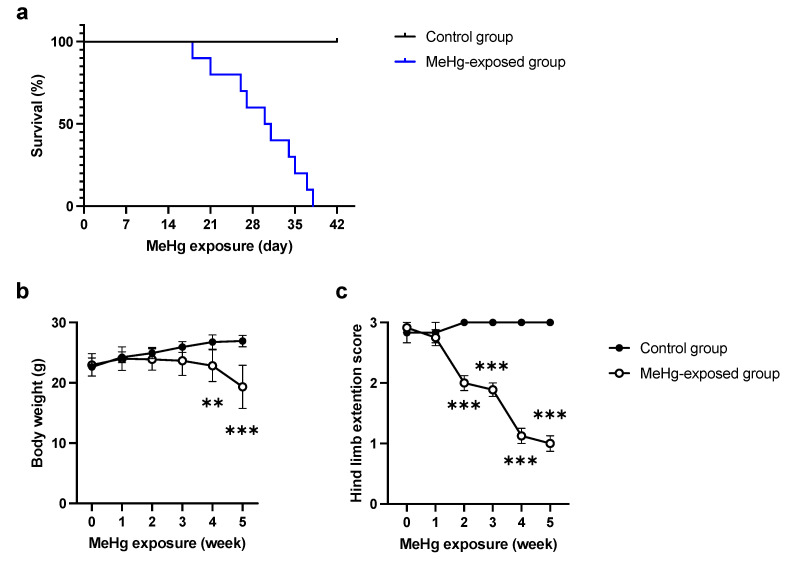
Sensitivity to MeHg exposure in ERAI-Venus mice in vivo. Mortality (**a**), body weight (**b**), and hind limb extension (**c**) in MeHg-exposed ERAI-Venus mice. MeHg-exposed mice were administered 50 ppm MeHg via drinking water. Data are expressed as the mean ± S.E.M. values (n = 10, ** *p* < 0.01, and *** *p* < 0.001: significant difference compared with control mice by two-way ANOVA with Tukey’s post hoc test).

First, we investigated the sensitivity of ERAI-Venus mice to MeHg. We examined the survival rate of MeHg-exposed ERAI-Venus mice and observed a decrease in the survival rate of the MeHg-exposed group in the late phase of MeHg exposure (Figure 1a). In addition, a reduction in body weight was observed (Figure 1b). To investigate whether motor dysfunction, one of the neurological symptoms of MeHg intoxication [20], is observed in MeHg-exposed ERAI-Venus mice, we examined changes in hind-limb extension. The results showed that hindlimb extension was suppressed after 2 weeks of exposure and tended to worsen over time (Figure 1c). These results were not significantly different from those of wild-type mice (Figure S1a–c), suggesting that ERAI-Venus mice can reflect central nervous system (CNS) pathology caused by MeHg intoxication in wild-type mice.

### 2.2. ER Stress Induction by MeHg Exposure in the Cerebral Cortex and Striatum

**Figure 2:**
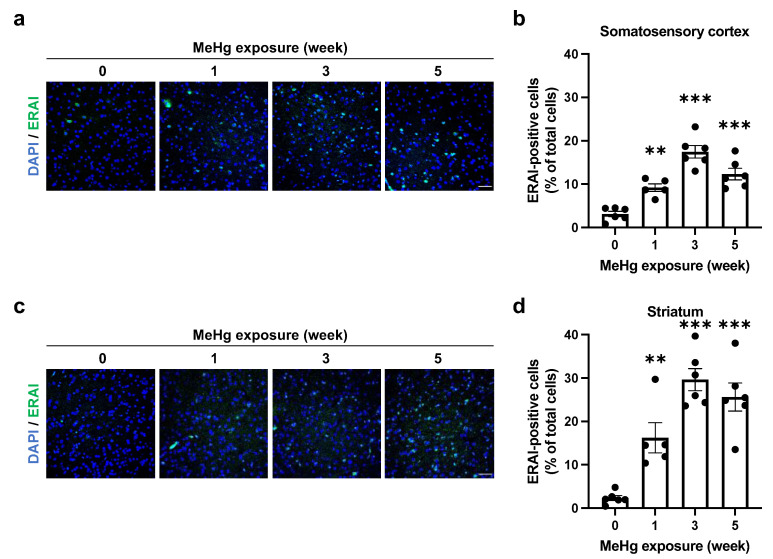
Time course analysis of MeHg-induced ER stress using ERAI-Venus mice. ERAI-Venus mice were exposed to 50 ppm MeHg via drinking water. At the indicated times, the mice were sacrificed, and the brain tissues were analyzed by immunostaining. (**a**,**c**) ERAI fluorescence signal in the somatosensory cortex and striatum of ERAI-Venus mice exposed to MeHg for the indicated time. The scale bar represents 50 μm. (**b**,**d**) Quantification of ERAI-positive cells shown in (**a**,**c**). Data are expressed as the mean ± S.E.M. values (n = 5–6, ** *p* < 0.01, and *** *p* < 0.001: significant difference compared with control mice without MeHg exposure (0 weeks) by one-way ANOVA with Dunnett’s post hoc test).

In MeHg-exposed mice, it has been reported that neurons in the cerebral cortex drop out [4,5]. In addition, MeHg has been reported to damage the striatum of mice [21]. However, few reports have demonstrated differences in MeHg sensitivity among these regions. We have previously shown that ER stress is elicited in a neuron-specific manner in the damaged regions by MeHg (somatosensory cortex, motor cortex, visual cortex, auditory cortex, and striatum), but the sensitivity differs among these regions [22]. In a previous study, the site-specific toxicity of MeHg was indicated to arise from the expression level of antioxidant proteins [23]. Therefore, we predict that other factors, such as the levels of antioxidant proteins, are involved in the site-specific ERAI signal induction. Common neurological symptoms of MeHg intoxication include sensory and motor dysfunction [20]. Therefore, we focused on the somatosensory cortex and striatum to investigate changes in ER stress induction upon exposure to 50 ppm MeHg. First, we analyzed mercury accumulation in the somatosensory cortex and striatum. We found a time-dependent increasing trend in both regions (Figure S2a,b). We then examined ERAI signaling in each region and found that ER stress was induced in both regions with a peak at 3 weeks after MeHg exposure (Figure 2a–d).

### 2.3. The UPR Signals Are Altered by MeHg Exposure in the Mouse Brain

**Figure 3:**
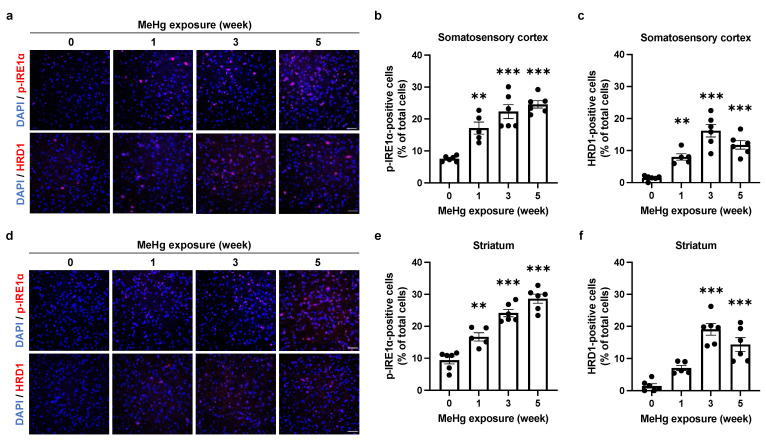
Effect of MeHg exposure on IRE1α-XBP1 axis in the mouse brain. (**a**,**d**) Detection of p-IRE1α and HRD1 in the somatosensory cortex (**a**) or striatum (**d**) of ERAI-Venus mice exposed to MeHg for the indicated times. Each scale bar represents 50 μm. (**b**,**e**) Quantification of p-IRE1α-positive cells in the somatosensory cortex (**b**) or striatum (**e**). (**c**,**f**) Quantification of HRD1-positive cells in the somatosensory cortex (**c**) or striatum (**f**). Data are expressed as the mean ± S.E.M. values (n = 5–6, ** *p* < 0.01, and *** *p* < 0.001: significant difference compared with control mice without MeHg exposure (0 weeks) by one-way ANOVA with Dunnett’s post hoc test).

Upon sensing ER stress, IRE1α is activated via autophosphorylation and induces the expression of molecular chaperones and ERAD-related proteins by splicing XBP1, thereby relieving ER stress [24,25]. We have previously shown that 30 ppm MeHg exposure transiently upregulates the expression of HRD1, a downstream factor of the IRE1α-XBP1 axis, similar to the ERAI signaling pattern [22]. Therefore, we examined the changes in IRE1α activity and downstream HRD1 expression by 50 ppm MeHg exposure. In the somatosensory cortex (Figure 3a–c) and striatum (Figure 3d–f), there was a trend toward a time-dependent increase in the number of phosphorylated IRE1α (p-IRE1α)-positive cells upon MeHg exposure, but the increase in HRD1-positive cells was only transient. Previously, we confirmed that XBP1 mRNA splicing is reduced and apoptosis is promoted via S-mercuration of IRE1α in vitro [16]. Since XBP1 is involved as a transcription factor in inducing the expression of HRD1, a downstream factor in the IRE1α pathway, it is possible that IRE1α function may be altered by S-mercuration in the mouse brain.

**Figure 4:**
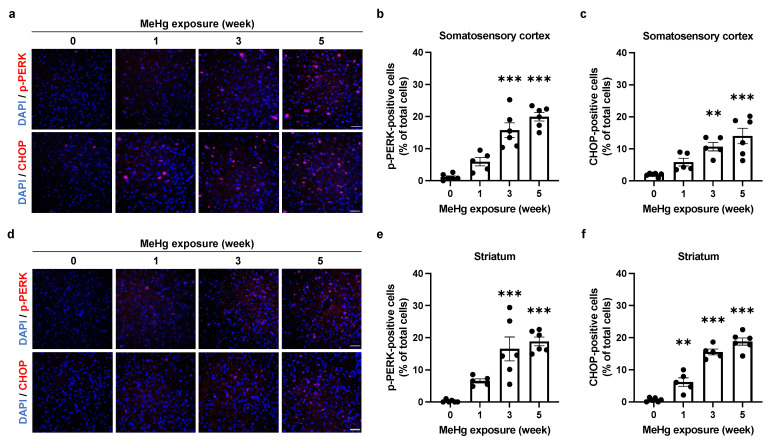
Effect of MeHg exposure on PERK branch in the mouse brain. (**a**,**d**) Detection of p-PERK and CHOP in the somatosensory cortex (**a**) or striatum (**d**) of ERAI-Venus mice exposed to MeHg for the indicated times. Each scale bar represents 50 μm. (**b**,**e**) Quantification of p-PERK-positive cells in the somatosensory cortex (**b**) or striatum (**e**). (**c**,**f**) Quantification of CHOP-positive cells in the somatosensory cortex (**c**) or striatum (**f**). Data are expressed as the mean ± S.E.M. values (n = 5–6, ** *p* < 0.01, and *** *p* < 0.001: significant difference compared with control mice without MeHg exposure (0 weeks) by one-way ANOVA with Dunnett’s post hoc test).

When ER stress is not resolved, cells induce expression of the ER stress-mediated apoptosis-inducing factor, CHOP, via the PERK or ATF6 pathway, thereby promoting apoptosis [26]. We have previously shown that the number of CHOP-positive cells increases in a time-dependent manner in neurons [22]. Therefore, to clarify the contribution of ER stress and the PERK pathway to MeHg-induced neuronal cell death, we investigated the time-dependent induction of CHOP expression and activation of the PERK branch. We detected a time-dependent increase in the number of phosphorylated PERK (p-PERK)- and CHOP-positive cells, suggesting that the UPR is induced toward apoptosis at the site of MeHg damage (Figure 4a–f).

### 2.4. Alterations of UPR Signaling by MeHg Are Related to Cell Death

**Figure 5:**
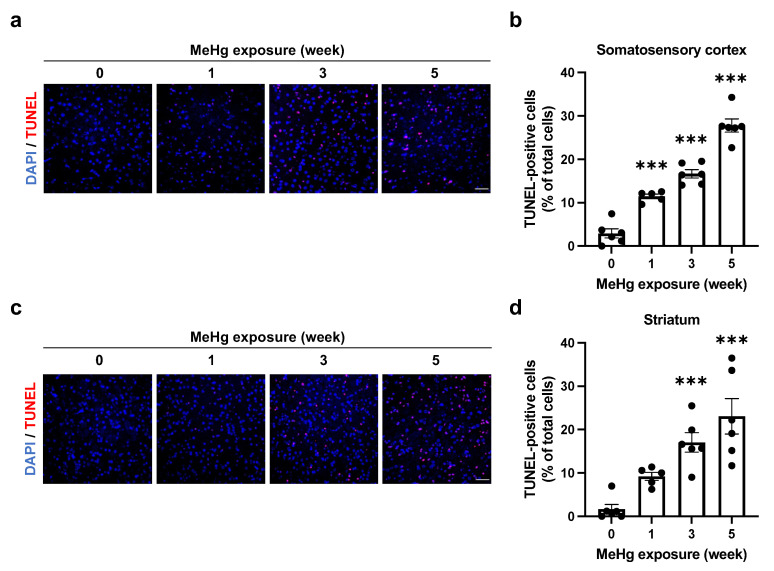
Induction of cell death in the mouse brain by MeHg exposure. (**a**,**c**) Detection of TUNEL-positive cells in the somatosensory cortex (**a**) or striatum (**c**) of ERAI-Venus mice exposed to 50 ppm MeHg for the indicated times. Each scale bar represents 50 μm. (**b**,**d**) Quantification of TUNEL-positive cells shown in (**a**,**c**), respectively. Data are expressed as the mean ± S.E.M. values (n = 5–6, *** *p* < 0.001: significant difference compared with control mice without MeHg exposure (0 weeks) by one-way ANOVA with Dunnett’s post hoc test).

To investigate whether alterations in UPR signaling caused by MeHg exposure are related to neuronal cell death, we detected apoptotic cells by using TUNEL staining. After 3 weeks of treatment, 30 ppm MeHg-exposed mouse brain showed TUNEL-positive cells and a significant increase 6 weeks after treatment (Figure S4a,b). Furthermore, an increase in TUNEL-positive cells in the somatosensory cortex was observed 1 week after 50 ppm MeHg administration, and the increase was time-dependent (Figure 5a,b). This was also observed in the striatum (Figure 5c,d). We then investigated whether the apoptotic cells resulted from a UPR-mediated cell death mechanism. The correlation between apoptosis-inducing factor CHOP or ERAI signaling and apoptosis was analyzed by immunohistochemistry and TUNEL staining in the somatosensory cortex of mice exposed to 30 ppm MeHg for 6 weeks. As a result, most CHOP-expressing cells were TUNEL-positive, while the TUNEL-positive rate of ERAI-signaling cells was low (Figure S5a,b). The low TUNEL-positive rate of ERAI-signaling cells may be due to the signal being reduced by the progression of apoptosis. If CHOP induction is caused by ER stress, co-localization of ERAI signaling and CHOP should be seen earlier than the 6-month exposure. As we predicted, we detected CHOP- and ERAI-positive cells in the somatosensory cortex of mice exposed to 30 ppm MeHg for 3 weeks by immunohistochemistry and found that they were co-localized (Figure S5c). These results suggest that MeHg-mediated ER stress induction and activation of the UPR induce cell death in the mouse brain.

## 3. Discussion

The induction of ER stress by MeHg exposure is thought to be involved in neuronal cell death. However, it was unclear how MeHg affects the UPR, which is responsible for both “cell survival” and “cell death” functions. In this study, we exposed MeHg to ERAI-Venus mice using the ERAI system and observed neurological symptoms and ER stress (Figure 1 and Figure 2). We noted that cytoprotective signaling by the IRE1α-XBP1 pathway and the activation of apoptotic signaling by the PERK and ATF6 pathways were differentially activated in the regions where MeHg-induced injury has been reported (cerebral cortex and striatum). The pathway peaked at 3 weeks of MeHg exposure and then decayed. In contrast, a time-dependent activation of the PERK pathway was observed (Figure 3 and Figure 4). These results suggest that MeHg accumulation in the mouse brain shifts the UPR signaling from cell survival to cell death, resulting in neuronal apoptosis. Indeed, an increase in apoptotic cells was observed in the mouse brain during the late phase of MeHg exposure (Figure 5). We have previously shown that the ERAI signal was observed predominantly in NeuN-positive neurons in the somatosensory cortex [22], suggesting that MeHg-mediated ER stress induction and alterations in UPR signaling are involved in neuronal cell death. 

Reactive electrophile species (RES) such as MeHg and NO can attract to bond to nucleophiles, which are molecules with the property of supplying electron pairs [27]. Reactive electrophile species can regulate physiological conditions that are essential for maintaining homeostasis. However, a pathological state is formed by disruption of the balance of these mechanisms. Malignant neoplasms, diabetes mellitus, and neurodegenerative diseases, including AD, PD, and amyotrophic lateral sclerosis (ALS), have been reported to involve RES [28,29,30,31]. In addition, it has been gradually identified that modulation of ER stress by RES is a critical pathogenic event for these diseases [32,33]. We have previously reported that MeHg induces ER stress via S-mercuration of PDI [14]. In addition, we have also shown that nitric oxide (NO) induces ER stress via oxidative modification (S-nitrosylation) of PDI [17]. In this study, we used ERAI-Venus mice to observe the effects of high concentrations of MeHg on ER stress in vivo and to verify its correlation with cell death. From the establishment of this model, it is expected that the ERAI system will be applied to elucidate the involvement of endoplasmic reticulum stress induction by RES in the pathogenesis of disease.

Patients with MeHg toxicity, such as those with Minamata disease, exhibit neuronal damage in the cerebellum and cerebral cortex, such as the postcentral gyrus (somatosensory cortex), Heschl’s gyrus (auditory cortex), and calcarine sulcus (visual cortex) [4]. Additionally, MeHg-exposed mice have been reported to show damage to the motor cortex and striatum [5,21], suggesting that damage in these regions is associated with neurological symptoms such as motor and sensory dysfunction. However, it is unclear whether ERAI-Venus mice exhibit neurological symptoms such as motor and sensory dysfunction by MeHg exposure. In this study, we found that hind-limb extension was suppressed by 50 ppm MeHg exposure in a time-dependent manner (Figure 1c) and this result was comparable with wild-type mice (Figure S1c). In TUNEL staining, the number of TUNEL-positive cells increased in a time-dependent manner (Figure 5), indicating that increased neuronal cell death due to MeHg exposure is responsible for the onset of neurological symptoms.

Next, we focused on the induction of ERAI signaling and activation of the UPR in the somatosensory cortex and striatum (Figure 2). To ameliorate ER stress, spliced XBP1 functions as a translocation factor inducing the expression of molecular chaperones and ERAD-related proteins [24,25]. We have previously shown that MeHg exposure attenuates the splicing of XBP1 mRNA by IRE1α in vitro [16]. Since the ERAI molecule is a fusion protein of XBP1 and the indicator Venus, a decrease in ERAI signaling implies a decrease in XBP1 mRNA splicing or degradation of the ERAI molecule. The activation of the IRE1α-XBP1 axis involved in the induction of ERAI signaling showed that phosphorylated IRE1α increased in a time-dependent manner, and expression of the downstream factor HRD1 peaked at 3 weeks after MeHg exposure, similar to the ERAI signaling (Figure 3). MeHg accumulates in the cortex and striatum (Figure S2a,b), suggesting that sustained exposure to MeHg may have inhibited XBP1 mRNA splicing and attenuated ERAI signaling and HRD1 expression. On the other hand, the number of phosphorylated PERK- and CHOP-positive cells increased in a time-dependent manner in the somatosensory cortex and striatum (Figure 4). It is believed that survival and cell death signals are simultaneously activated in the UPR, and that the weakening of survival signals leads to the predominance of cell death signals and induces apoptosis [34]. Therefore, these findings suggest that cells reduce ER stress via the IRE1a-XBP1 pathway in short-term MeHg exposure, but with sustained MeHg exposure, splicing XBP1 reduction and activation of the PERK and ATF6 pathways induce apoptosis via expression of the apoptosis-inducing factor CHOP. However, it is necessary to prove in more detail the involvement between MeHg-induced neuronal cell death and changes in the UPR activation. Several reports indicated that the activation of ASK1/JNK signaling and caspases are involved in the induction of apoptosis by MeHg exposure [6,35]. Hence, the activation of these apoptosis pathways should also be investigated. In addition, we expect that the MeHg-induced neuronal cell death mechanism will be elucidated by conducting comprehensive transcriptome analysis of the activating factors in each signal and by conducting analysis using inhibitors of ER stress and UPR activation.

In conclusion, ERAI-Venus mice reflect the neurological damage caused by MeHg exposure, and it is possible to evaluate the selective toxicity caused by MeHg. Furthermore, we found that MeHg-induced ER stress contributes to cell death by inducing UPR signaling changes at MeHg-impaired sites in the brain. These findings suggest that modulation of the UPR may be a therapeutic target for the treatment of neurological and other disorders caused by MeHg intoxication.

## 4. Materials and Methods

### 4.1. Animals

Male ERAI-Venus mice were generated as described previously [19] and maintained on a C57BL/6 background. C57BL6N/Jc1 mice were purchased from CLEA Japan (Tokyo, Japan) and housed in the National Institute for Minamata Disease. Seven-week-old ERAI-Venus mice were used for immunohistochemistry. 

### 4.2. MeHg Administration

ERAI-Venus mice were randomly divided into the control (n = 6) and MeHg-exposed groups (n = 5–6 mice/group). Mice were exposed to MeHg via drinking water containing 30 or 50 ppm MeHg as MeHg-GSH (FUJIFILM Wako Pure Chemical, Osaka, Japan) (1:1) complex as previously described [5]. ERAI-Venus mice in the control group were provided GSH-containing water.

### 4.3. Hind Limb Extension Analysis

To examine hind limb flaccidity by MeHg, mice were gently removed from their home cage and suspended by the tail for ten seconds. It was monitored once a week during the 35 days of MeHg exposure and provided a hind limb extension score as follows: normal escape extension = 3, slightly incomplete splay and loss of mobility = 2, no splay and loss of mobility = 1, complete crossing of hind limbs = 0.

### 4.4. Measurement of Mercury Deposition

Measurement of mercury deposition was described previously [22]. ERAI-Venus mice were sacrificed at the indicated times, and the cerebral cortex and striatum were removed from their right brains. Tissues were dissolved with 5N NaOH (Nacalai Tesque, Kyoto, Japan) solution and boiled at 70 °C for 30 min. After neutralization with 5N HCl (Nacalai Tesque, Kyoto, Japan), the total concentration of mercury was measured by the oxygen combustion-gold amalgamation method using an MA2000 analyzer (Nippon Instruments Corporation, Tokyo, Japan).

### 4.5. Immunohistochemistry

ERAI-Venus mice were sacrificed, and their left brains were extracted and fixed with 4% paraformaldehyde (FUJIFILM Wako Pure Chemical, Osaka, Japan) in PBS. After embedding in paraffin, the brains were sliced into 5 µm coronal sections using microtome. Paraffin-embedded sections were dewaxed with xylenes, rehydrated through successive ethanol washes and boiled for 20 min in pH 6.0 citrate buffer (LSI Medience, Tokyo, Japan) for antigen retrieval. Immunofluorescence staining was performed using an M.O.M. immunodetection kit (#FMK-2201, Vector Laboratories, Burlingame, CA, USA) with the following antibodies: anti-green fluorescent protein (GFP) (1:200; #M048-3, Medical & Biological Laboratories, Nagoya, Japan) and anti-CHOP (1:1000; #ab11419, Abcam, Cambridge, UK). Anti-HMG-CoA reductase degradation enzyme 1 (HRD1) (1:500; #sc-130889, Santa Cruz Biotechnology, Dallas, TX, USA), anti-p-IRE1α (1:1200; #ab48187, Abcam, Cambridge, UK) and anti-p-PERK (1:2000; #sc-32577, Santa Cruz Biotechnology, Dallas, TX, USA) antibodies were diluted in PBS with 5% BSA and incubated overnight at 4 °C. After one wash with PBS, a goat anti-rabbit IgG Alexa Fluor 594 (1:200; #A-11012, Thermo Fisher Scientific, Waltham, MA, USA) secondary antibody was applied and incubated for one hour at room temperature. Nuclei were stained with a mountant containing DAPI (Thermo Fisher Scientific, Waltham, MA, USA). All images were acquired with an ECLIPSE Ti microscope (Nikon, Tokyo, Japan) and analyzed with ImageJ software (NIH, Bethesda, MD, USA).

### 4.6. TUNEL Staining

In Situ Cell Death Detection Kit, TMR red (12156792910, Roche, Basel, Switzerland) was used to observe cell apoptosis under a confocal fluorescence microscope. Paraffin-embedded sections were dewaxed, rehydrated, and antigen-activated as described in Section 4.5. Then, 5%; BSA diluted in PBS was added to the sections and incubated for one hour at room temperature. The sections were incubated with TUNEL reaction solution for one hour at 37 °C. After washing with PBS, the sections were sealed with DAPI staining the nuclei.

### 4.7. Statistical Analysis

All results are expressed as the mean ± S.E.M. values. Statistical comparisons were performed by one-way or two-way ANOVA followed by Dunnett’s or Tukey’s test. All data were analyzed with GraphPad Prism 9 (GraphPad Software, San Diego, CA, USA). Statistical significance is represented as * *p* < 0.05, ** *p* < 0.01, *** *p* < 0.001. The dots in the graph in Figure 2, Figure 3, Figure 4 and Figure 5 represent the analyzed values for each trial.

## Supplementary Materials

The following are available online at https://www.mdpi.com/article/10.3390/ijms232315412/s1, Figure S1: Sensitivity to MeHg exposure in wild-type mice in vivo., Figure S2: The amount of mercury in the mouse brain., Figure S3: Effect of MeHg exposure on each UPR branch in the cerebral cortex., Figure S4: Induction of cell death in the mouse brain by 30 ppm MeHg exposure., Figure S5: Association between alterations in UPR signalings and neuronal cell death.
