# Supplementary material for: Alterations in UPR Signaling by Methylmercury Trigger Neuronal Cell Death in the Mouse Brain
Source: Int J Mol Sci. 2022 Dec 6;23(23):15412. doi: 10.3390/ijms232315412 (PMC9738736; doi:10.3390/ijms232315412)
Supplement: Supplementary file 1 [file ijms-23-15412-s001.zip › ijms-2053465-supplementary.pdf]

International Journal of Molecular Science

"Alterations in UPR signaling by methylmercury trigger neuronal cell death in the mouse brain"

Ryosuke Nomura [1], Nobumasa Takasugi [1], Hideki Hiraoka [1], Yuta Iijima [1], Takao Iwawaki [2], Yoshito Kumagai [3], Masatake Fujimura [4*] and Takashi Uehara [1*]

Supplemental Materials

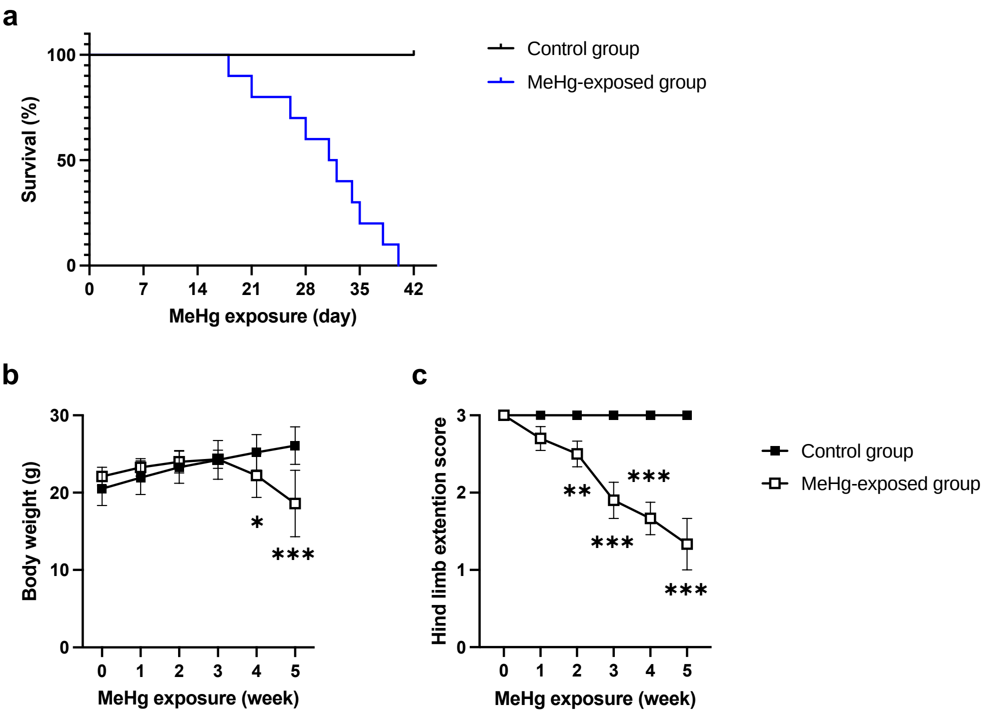

Figure S1. Sensitivity to MeHg exposure in wild-type mice in vivo. Mortality (**a**), body weight (**b**) and hind limb extension (**c**) in control or MeHg-exposed wild-type mice. MeHg-exposed mice were administrated 50 ppm MeHg via drinking water. Data are expressed as the mean ± S.E.M. values (n = 10, $*p < 0.05$, $**p < 0.01$ and $***p < 0.001$: significant difference compared with control mice by two-way ANOVA with Tukey's post hoc test).

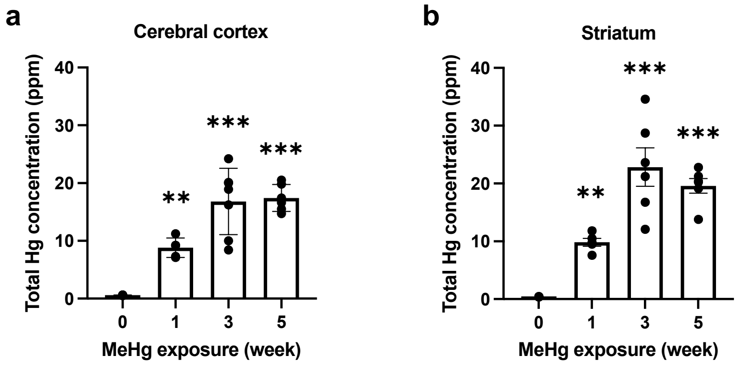

Figure S2. The amount of mercury in the mouse brain. (**a**), (**b**) ERAI-Venus mice were exposed to 50 ppm MeHg via drinking water. At the indicated times, the mice were sacrificed, and the cerebral cortex and striatum were used for the measurement of total mercury concentration. Data are expressed as the mean ± S.E.M. values (n = 5–6, $**p < 0.01$ and $***p < 0.001$: significant difference compared with mice without MeHg exposure (0 weeks) by one-way ANOVA with Dunnett's post hoc test).

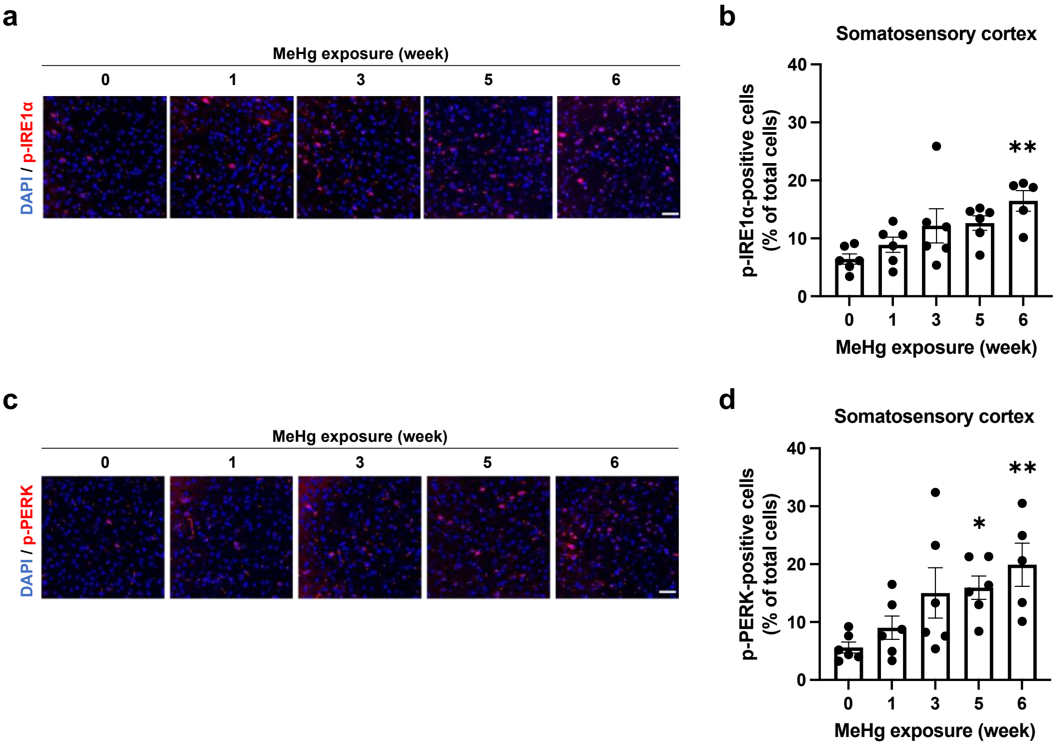

Figure S3. Effect of MeHg exposure on each UPR branch in the cerebral cortex. (**a**), (**c**) Detection of p-IRE1α (a) and p-PERK (b) in the somatosensory cortex of ERAI-Venus mice exposed to 30 ppm MeHg for the indicated times. Each scale bar represents 50 µm. (**b**), (**d**) Quantification of p-IRE1α- and p-PERK-positive cells in the somatosensory cortex (a), (b), respectively. Data are expressed as the mean ± S.E.M. values (n = 5–6, **p* < 0.05 and ***p* < 0.01: significant difference compared with mice without MeHg exposure (0 weeks) by one-way ANOVA with Dunnett's post hoc test)

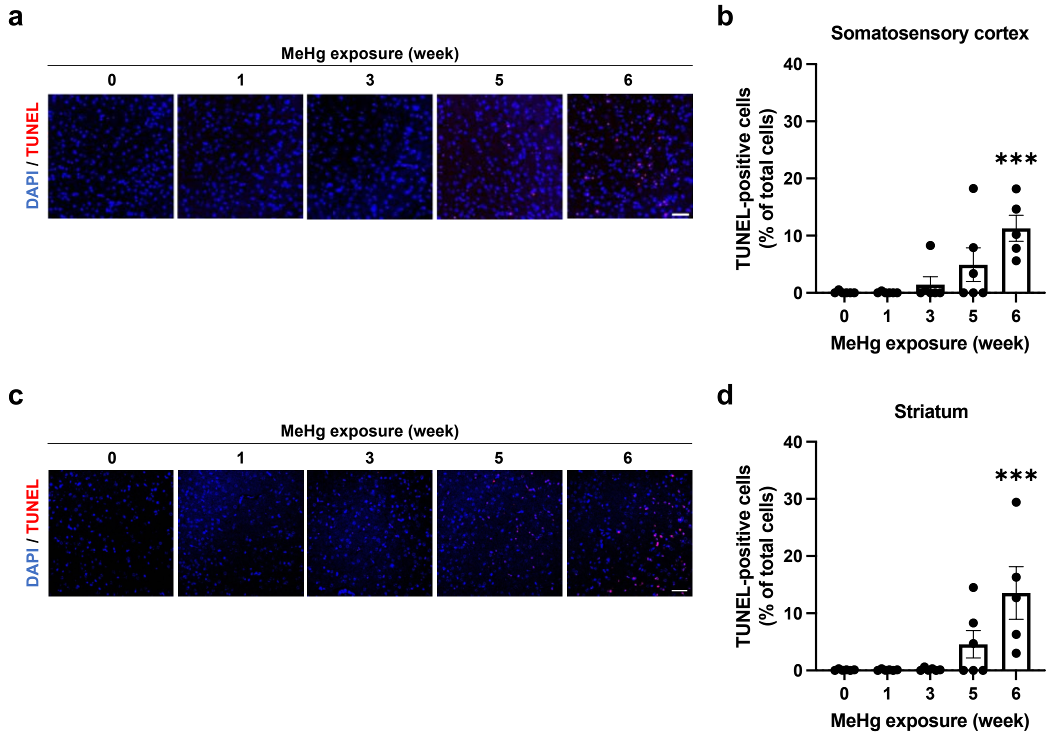

Figure S4. Induction of cell death in the mouse brain by 30 ppm MeHg exposure. (**a**), (**c**) Detection of TUNEL in the somatosensory cortex (a) or striatum (c) of ERAI-Venus mice exposed to 30 ppm MeHg for the indicated times. Each scale

bar represents 50 μm. (**b**), (**d**) Quantification of TUNEL-positive cells shown in (a) and (c), respectively. Data are expressed as the mean ± S.E.M. values (n = 5–6, ****p* < 0.001: significant difference compared with mice without MeHg exposure (0 weeks) by one-way ANOVA with Dunnett's post hoc test)

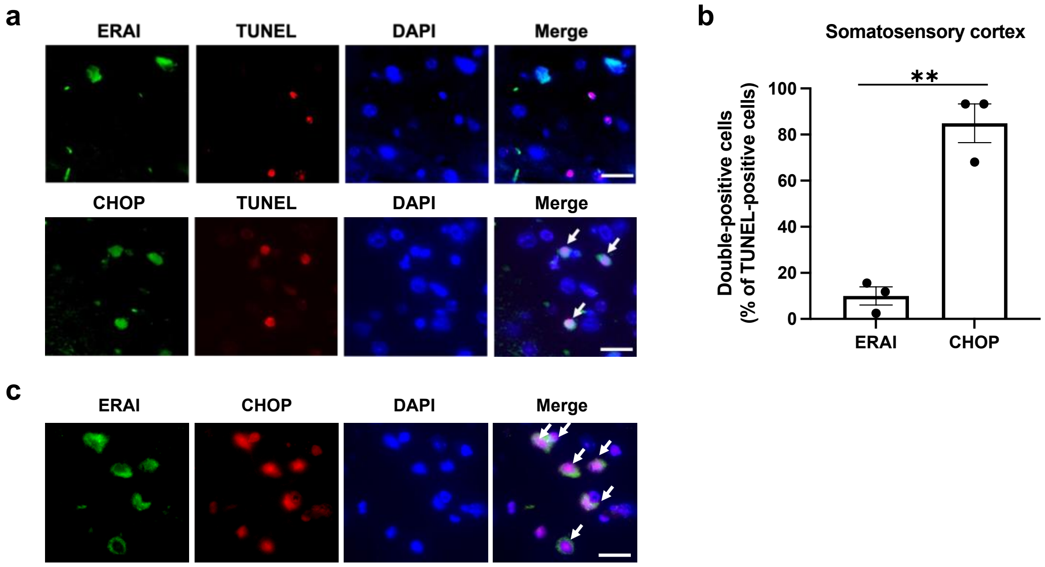

Figure S5. Association between alterations in UPR signalings and neuronal cell death. (**a**) Detection of the ERAI signal or CHOP (green) in cells positive for the apoptosis marker TUNEL (red) in the somatosensory cortex of ERAI-Venus mice exposed to 30 ppm MeHg for 6 weeks. Each scale bar represents 20 μm. The arrows indicate the cells colocalizing CHOP and TUNEL. (**b**) Quantification of cells coexpressing each signal and TUNEL in (a). Data are expressed as the mean ± S.E.M. values (n = 3, ** p < 0.01 by Student's t-test). (**c**) Coexpression of ERAI signal (green) and CHOP (red) in the somatosensory cortex of ERAI-Venus mice exposed to 30 ppm MeHg for 3 weeks. the scale bar represents 20 μm. The arrows indicate the cells coexpressing ERAI signal and CHOP.
